# Chiral Hydroxy Metabolite of Mebendazole: Analytical and Semi-Preparative High-Performance Liquid Chromatography Resolution and Chiroptical Properties

**DOI:** 10.3390/ph17060696

**Published:** 2024-05-28

**Authors:** Paolo Guglielmi, Gaia Pulitelli, Francesca Arrighi, Daniela Secci, Marco Pierini, Roberto Cirilli

**Affiliations:** 1 Department of Chemistry and Technology of Drugs, Sapienza University of Rome, 00185 Rome, Italy; paolo.guglielmi@uniroma1.it (P.G.); francesca.arrighi@uniroma1.it (F.A.); daniela.secci@uniroma1.it (D.S.) marco.pierini@uniroma1.it (M.P.); 2 National Centre for the Control and Evaluation of Medicines, Chemical Medicines Unit, Istituto Superiore di Sanità, Viale Regina Elena 299, 00161 Rome, Italy

**Keywords:** hydroxy mebendazole, Chiralpak IG, enantioselective HPLC, chiroptical properties, theoretical calculations

## Abstract

Mebendazole (**MBZ**) is a benzimidazole carbamate anthelmintic used worldwide for the treatment and prevention of parasitic disorders in animals and humans. A large number of in vivo and in vitro studies have demonstrated that **MBZ** also has anticancer activity in multiple types of cancers. After oral administration, the phenylketone moiety of **MBZ** is rapidly reduced to the hydroxyl group to form the chiral hydroxy metabolite (**MBZ-OH**). To the best of our knowledge, there is no information in the literature on the stereochemical course of transformation and the anthelmintic and antitumor activity of individual enantiomers of **MBZ-OH**. In the present study, we describe in detail the direct HPLC resolution of **MBZ-OH** on a 100 mm × 4.6 mm Chirapak IG-3 column packed with 3 μm silica particles containing amylose (3-chloro-5-methylphenylcarbamate) as a selector. At 25 °C and using pure methanol as the mobile phase, the enantioseparation and resolution factors were 2.38 and 6.13, respectively. These conditions were scaled up at a semi-preparative scale using a 250 mm × 10 mm Chiralpak IG column to isolate multi-milligram amounts of both enantiomeric forms of the chiral metabolite. The chiroptical properties of the collected enantiomers were determined and, through a theoretical study, were related to the more stable conformations of MBZ-OH. The first and second eluted enantiomers were dextrorotatory and levorotatory, respectively, in dimethylformamide solution. Finally, by recording the retention factors of the enantiomers as the water content in the water–acetonitrile mobile phases was progressively varied, U-shaped retention maps were generated, indicating a dual and competitive hydrophilic interaction liquid chromatography and reversed-phase liquid chromatography retention mechanism on the Chirapak IG-3 chiral stationary phase.

## 1. Introduction

**Figure 1:**
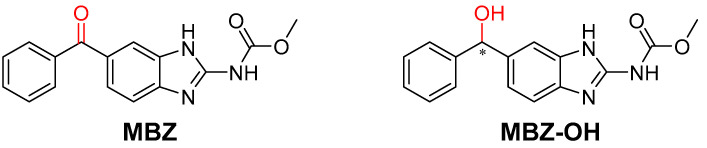
Structures of mebendazole (**MBZ**) and reduced mebendazole (**MBZ-OH**).

Mebendazole (**MBZ**) (methyl-5-benzoyl-2-benzimidazole carbamate) (Figure 1) is a benzimidazole carbamate that is widely used in human and veterinary medicine for the treatment of a variety of worm infestations [1]. Over the past decade, a large number of in vitro studies have demonstrated that **MBZ** also has anticancer activity in several types of cancer, including adrenocortical carcinoma, gastric cancer, colorectal cancer, non-small cell lung cancer, melanoma, and ovarian and glioblastoma multiforme [2]. **MBZ** has been observed to induce growth arrest and apoptosis in cultured cancer cells at doses that have minimal effect on non-cancerous cells [3,4].

In human cells, **MBZ** inhibits the formation of a microtubule-based organelle that acts as a signaling hub for triggering the Hedgehog (Hh) pathway, thereby interfering with tubulin polymerization. **MBZ** binds tubulin at a site that is also recognized by colchicine [5]. In addition, it has low toxicity and is well tolerated, even at high doses over long periods. For these reasons, and because the development, screening, preclinical testing and clinical trials of new anticancer compounds are costly and time-consuming, **MBZ** is being studied extensively as a potential anticancer agent, either alone or in combination with existing anticancer drugs [6,7,8].

The use of **MBZ** in the so-called drug repurposing process in oncology may offer interesting advantages. It is a well-characterized drug and we have extensive knowledge of its pharmacodynamics, pharmacokinetics and side effects, as well as established protocols for clinical use.

One of the major plasma metabolites of **MBZ** is the hydroxy reduced metabolite [methyl (6-(hydroxy(phenyl)methyl)-1*H*-benzo[*d*]imidazol-2-yl)carbamate)] (**MBZ-OH**, Figure 1), which is mainly glucuronidated and excreted via the bile. This metabolite has less anthelmintic activity than **MBZ [9]**. Information is lacking on the enantioselectivity of the reduction process from **MBZ** to **MBZ-OH** as well as on the biological activity, including antitumor activity, of the individual enantiomers. A more specific understanding of **MBZ** metabolism and the enzymatic pathways involved in the in vivo reduction in the phenyl ketone moiety should improve our understanding of the factors that influence its anthelmintic and antitumor activity.

Biological systems are known to recognize the two enantiomers of a chiral compound as two distinct substances. The role of the three-dimensional structure in the discrimination process at the enzyme and receptor levels has led to the search for effective methods for the separation of enantiomers of a chiral drug candidate or metabolite and the study of their stereochemistry [10].

High-performance liquid chromatography (HPLC) on polysaccharide-based chiral stationary phases (CSPs) is the most commonly used technique for the direct resolution of racemic samples at analytical and preparative scales.

The availability of a wide range of polysaccharide chiral selectors for HPLC allows the successful selection of the best analytical conditions for the analytical resolution of a variety of chiral compounds under multimodal elution conditions [11,12,13,14,15]. The optimized enantioselective method can be easily scaled up to a semi-preparative scale to provide mg quantities of single enantiopure forms for biological testing and stereochemical characterization.

This work aims to develop an enantioselective HPLC method potentially able to (a) quantify the enantiomeric composition of biological samples containing **MBZ-OH** and thus support pharmacokinetic and pharmacodynamic studies on the reduced metabolite of **MBZ**, and (b) isolate enantiopure forms of **MBZ-OH** in amounts useful for biological assays and stereochemical characterization. The retention and enantioselective properties of the amylose-based Chiralpak IG-3 CSP towards **MBZ-OH** were investigated using polar organic and organic–aqueous eluent conditions.

Finally, the possibility of correlating the experimental chiroptical properties of the isolated enantiomers of **MBZ-OH** with those evaluated through a theoretical approach, to obtain a reasonably reliable assignment of the absolute configuration, has been carefully considered [16].

## 2. Results and Discussion

### 2.1. Synthesis and Chemical Characterization of ***MBZ-OH***

**Scheme 1:**
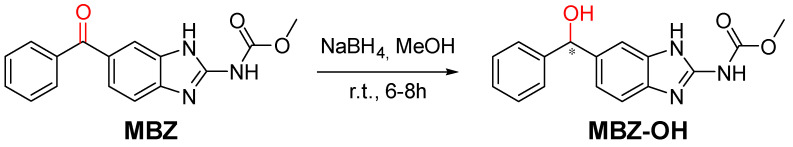
Synthesis of reduced mebendazole (**MBZ-OH**).

The synthesis of **MBZ-OH** was performed by reacting **MBZ** with an excess of sodium borohydride (NaBH_4_), a mild reducing agent employed for the selective reduction of aldehyde and ketones to alcohols (Scheme 1). The reaction was performed until completion (6 h to 8 h) in methanol at room temperature. 

**Figure 2:**
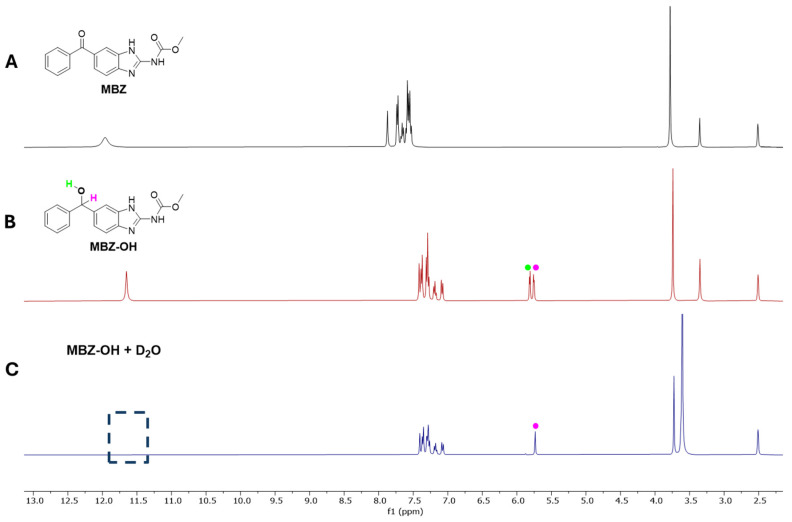
^1^H-NMR spectra comparison between **MBZ** and **MBZ-OH**. (**A**) ^1^H-NMR spectra of MBZ dissolved in DMSO-*d*_6_. (**B**) ^1^H-NMR spectra of MBZ-OH dissolved in DMSO-*d*_6_. (**C**) ^1^H-NMR spectra of MBZ dissolved in DMSO-*d*_6_ added by D_2_O. Green and pink circles define the signals attributable to the secondary alcohol obtained from reduction. The blue dashed rectangle indicates missing signals due to NH exchangeable protons.

**Figure 3:**
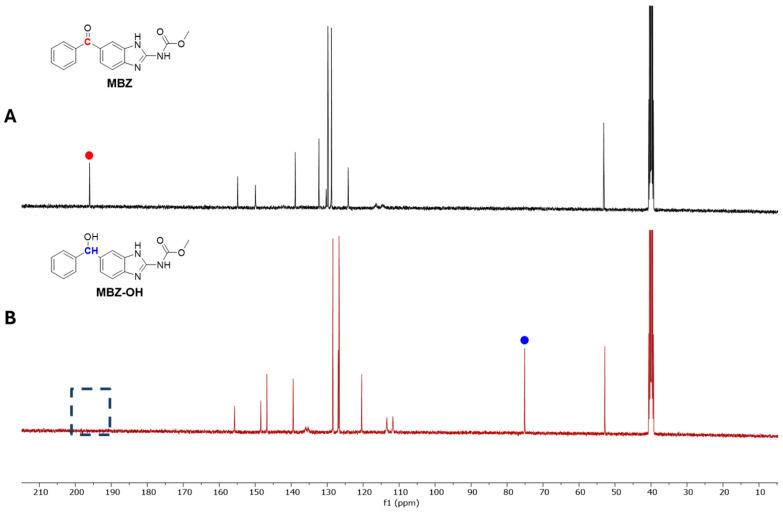
^13^C-NMR spectra comparison between **MBZ** (**A**) and **MBZ-OH** (**B**). The blue dashed rectangle indicates the disappearance of the mebendazole C=O signal, while the blue circle underlies the presence of a novel signal related to secondary alcohol obtained from reduction. Red circle shows the carbon signal that will disappear after conversion. Some signals of **MBZ** are absent or poorly visible due to the long relaxation times of quaternary carbons along with further broadening due to their proximity to nitrogen.

Upon purification through chromatography (for major details, see Section 3), the compound was characterized by means of ^1^H-NMR and ^13^C-NMR analysis (Figure 2 and Figure 3, respectively). Figure 2 reports the ^1^H-NMR spectra comparison between **MBZ** and **MBZ-OH** dissolved in deuterated dimethyl sulfoxide (DMSO-*d*_6_). Moreover, to unravel exchangeable protons ^1^H-NMR spectra were further acquired after the addition of deuterated water (D_2_O). The reduction in carbonyl moiety was confirmed by the appearance of the two doublets between 5.5 and 6.0 ppm, ascribed to the secondary alcohol system R^2^CH-OH (Figure 2B, green circle). As a matter of fact, the addition of D_2_O led to the disappearance of one of the novel doublets owing to exchange with the OH group (Figure 2C, absence of green circle). Similarly, the singlet at ≈11.5 ppm related to the NH signals (Figure 2C, blue dashed rectangle) was no longer visible after the addition of D_2_O. 

The comparison between the ^13^C-NMR spectra of **MBZ** and **MBZ-OH** also evidenced the reduction in the **MBZ** carbonyl moiety, demonstrated by the disappearance of the carbonyl signal at ≈196 ppm (Figure 3A, red ball) and the appearance of a novel aliphatic signal at ≈75 ppm (Figure 3B, blue circle) due to the presence of the secondary alcohol.

### 2.2. Analytical HPLC Enantioseparation under Polar Organic Conditions

Amylose (3-chloro-5-methylphenylcarbamate) (ACMPC) is known to be one of the most effective selectors for HPLC [11,12,17,18]. In the first version of an ACMPC-based CSP prepared by Okamoto et al. and commercialized, the polysaccharide was physically coated on 10 μm particles of macroporous silica gel. Although this CSP exhibited high enantioselectivity, due to its coated nature, it suffered from low versatility and stability and could only be used in combination with a limited range of solvents (typically mixtures of n-hexane and 2-propanol or ethanol) [17,18]. Currently, immobilized-type ACMPC-based chiral material packings for HPLC are commercially available [12,19,20,21,22]. In these types of CSPs, the polymer is anchored to the silica microparticles in such a way that it resists dissolution or imbibition by solvents typically used to prepare normal-phase eluents (dichloromethane, ethyl acetate, hydrocarbons, and short-chain alcohols).

In this work, the chiral discrimination of the 100 mm × 4.6 mm column packed with 3 μm particles of the ACMPC-based Chiralpak IG-3 CSP towards **MBZ-OH** was investigated using pure methanol and ethanol as mobile phases. The enantiorecognition ability of the Chiralpak IG-3 CSP was recorded at five different temperatures (i.e., 20, 25, 30, 35 and 40 °C).

Table 1 shows the chromatographic data obtained at a flow rate of 1 mL min^−1^.

At 20 °C: (i) the enantioseparation factor was high, around 2.5, for both alcohols; (ii) a slight increase in resolution from 4.84 to 5.79 was observed when switching from ethanol to methanol; (iii) retention factors were similar with the alcoholic eluents.

When the column temperature was changed in the range of 20–45 °C, retention and enantioselectivity decreased with increasing temperature in all cases.

Maximum resolution (*Rs* = 6.13) was obtained using methanol as the mobile phase and a column temperature of 25 °C. These analytical conditions were chosen to scale up the enantioseparation to a semi-preparative level.

By replacing the alcoholic eluent with acetonitrile at 25 °C, the enantiomers were not eluted after 30 min. This anomalous behavior of the column will be discussed in more detail in the next section.

### 2.3. Analytical HPLC Enantioseparation under Organic–Aqueous Conditions

**Figure 4:**
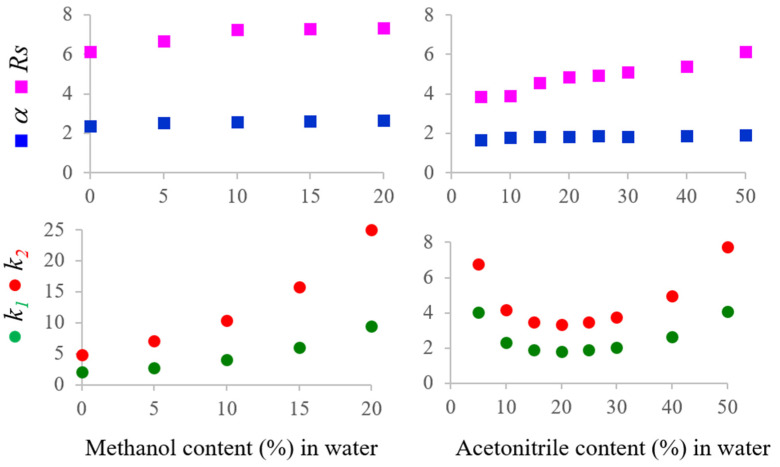
Plots of the retention (*k*_1_ and *k*_2_, green and red circles, respectively), enantioseparation (*α*, blue square) and resolution (*Rs*, magenta square) factors of **MBZ-OH** as a function of the water content in the methanol–water and acetonitrile–water mobile phases. Chromatographic conditions: column, Chiralpak IG-3 (100 mm × 4.6 mm, 3 μm); flow rate, 1 mL min^−1^; detection, UV at 220 nm.

Consistent with previous studies and taking into account the results obtained with acetonitrile as a mobile phase, it seems reasonable to hypothesize that strong polar sites responsible for the large retention of the enantiomers of **MBZ-OH** can operate in this elution mode. One way to uncover such interactions is to progressively add water to the mobile phase and record retention maps, namely *k* versus water % plots. Figure 4 shows the retention data obtained at 25 °C using binary mixtures containing acetonitrile at different percentages (i.e., 5%, 10%, 15%, 20%, 30%, 40% and 50%).

The first remarkable finding is that only a small percentage of water added to acetonitrile is required to overcome these attractive forces between complementary polar fractions of the stationary phase and the analyte and to elute the **MBZ-OH** enantiomers in a reasonable time. This tendency is typical of the occurrence of a predominant hydrophilic interaction liquid chromatography (HILIC) retention/separation mechanism in which water is the strongest eluting solvent, acting as a displacer, and acetonitrile is the weakest solvent [23,24,25]. The active sites of the CSP responsible for the enantioselective HILIC are unknown and are exposed using pure acetonitrile as the mobile phase. This allows strong and electrostatic intermolecular selector/selectand interactions to occur. Water molecules compete with the hydrophilic sites and dramatically reduce the retention of two enantiomers. The decrease is gradual with water increment until a critical mobile phase composition is reached (acetonitrile-water 80:20 *v*/*v*), beyond which the predominant mechanism of retention becomes typical of the reversed-phase (RP) mode. Therefore, the presence of water in the mobile phase determines a dualistic RP-like and HILIC-like behavior of Chiralpak IG-3 towards **MBZ-OH**. A clear indication of the dual and competitive HILIC-RP retention mechanism is the characteristic U-profile of the retention maps [26].

**Figure 5:**
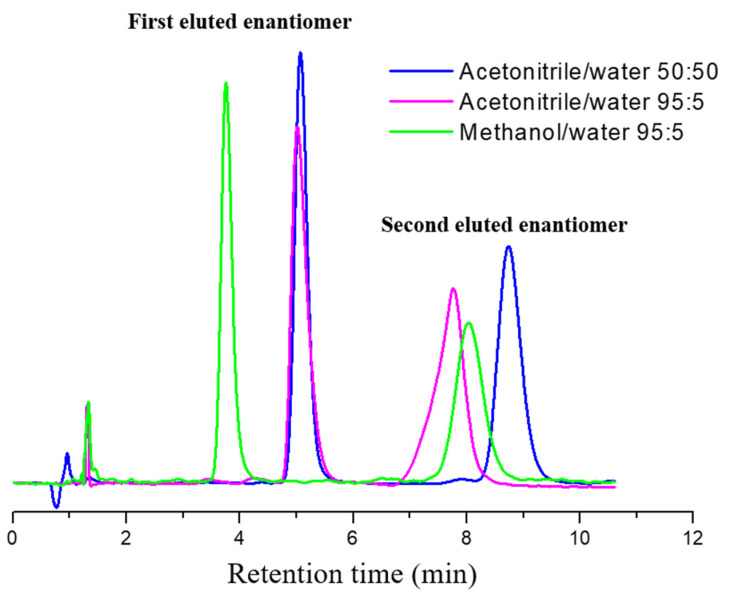
Typical chromatograms of **MBZ-OH** obtained in the methanol-aqueous and acetonitrile-aqueous modes. Chromatographic conditions: column, Chiralpak IG-3 (100 mm × 4.6 mm, 3 μm); mobile phases: acetonitrile–water 50:50 (blue line), acetonitrile–water 95:5 (magenta line) and methanol–water 95:5 (green line); flow rate, 1 mL min^−1^; detection, UV at 220 nm.

Figure 4 also shows the trend in enantioselectivity and resolution as a function of the incremental water content in acetonitrile. As can be seen, both chromatographic parameters are improved by the addition of water to the acetonitrile. Typical chromatograms illustrating this behavior are shown in Figure 5. The resolution of **MBZ-OH** under an HILIC-like elution mode (mobile phase: acetonitrile–water 95:5 *v*/*v*; *Rs* = 3.85) increases when passing under the RP domain (mobile phase: acetonitrile–water 95:5 *v*/*v*; *Rs* = 6.14) due to an improvement in the peak efficiency of the second eluting enantiomer and in the enantioselectivity (*α* = 1.69 vs. *α* = 1.90). Unlike acetonitrile, methanol provides hydrogen bond donor/acceptor interactions and can compete with water for the hydrophilic interactions. The impact on the trend of the retention map is clearly visible in Figure 4. The ability of methanol to form a water-like shielding film over the hydrophilic surface leads to a preferential interaction of the analytes with the stationary phase via hydrophobic interactions rather than HILIC interactions. The classical RP retention pattern of the retention map reflects the unidirectional mechanism. To compare the performance of the Chirapak IG-3 column under different elution modes, Figure 5 also shows the resolution of MBZ-OH obtained with the mixtures methanol–water 95:5 *v*/*v*, acetonitrile–water 95:5 *v*/*v* and acetonitrile–water 50:50 *v*/*v*. Under all these eluent conditions, the enantiomer separation was completed within 10 min. The best compromise between analysis time (i.e., 8 min) and resolution factor (*Rs* = 6.66) was obtained by using methanol–water 95:5 *v*/*v*.

Such versatility of the chiral support in interaction with **MBZ-OH** can be used to develop enantioselective methods for biological samples using eluents rich in the organic modifier, which is a favorable condition for LC/MS applications, or green eluents rich in water for more sustainable analysis.

### 2.4. Semi-Preparative Enantioseparation

The enantioselective HPLC approach is attractive to the pharmaceutical industry because it provides easy access to both enantiomers of chiral compounds in quantities suitable for preliminary biological comparisons, such as in vitro and in vivo tests and toxicological studies [27,28].

After selecting the best CSP-mobile phase system for analytical-scale enantiomer separation, the resolution must be transferred to a larger scale to isolate from a few milligrams to a few grams, which are typically sufficient quantities for full biological enantiomer characterization. By considering appropriate scale factors and using semi-preparative or preparative columns with the same packing material as the analytical columns, the scale-up process is usually easily accomplished, especially when the enantioseparation and resolution values are as high as those observed for **MBZ-OH** on the Chiralpak IG-3 CSP.

However, to design an effective multi-milligram or multigram separation, the solubility of the racemic sample in the mobile phase must be carefully considered.

The low solubility of a chiral sample in the mobile phase limits the amount of racemate that can be loaded onto the column in a single run [29]. As a result, the productivity of the HPLC enantioseparation may be greatly reduced or even compromised, even under conditions in which a high degree of enantioselectivity is observed.

In the case of **MBZ-OH**, the solubility in methanol, which is used as the only component of the mobile phase in the optimized enantioseparation method developed on the Chiralpak IG-3, is <1 mg mL^−1^. This problem could be solved by adding to the alcoholic eluent a solvent like dimethylformamide (DMF) in which **MBZ-OH** is highly soluble (i.e., >60 mg mL^−1^). The disadvantage of this choice is that the polar solvent has a high boiling point (153 °C) and therefore cannot be easily removed from the eluate by evaporation.

Therefore, to achieve the resolution of **MBZ-OH** on a semi-preparative scale, dimethylformamide (DMF) was used in a mixture with methanol to dissolve the racemic sample. Since the mismatch in the elution strength between the diluent and mobile phase can lead to aberrations in the chromatogram with unretained elution of part of the sample and splitting of enantiomeric peaks [30], samples containing the same mg of **MBZ-OH** in different DMF/methanol mixtures were prepared and injected on the semi-preparative 250 mm × 10 mm Chiralpak IG column.

**Figure 6:**
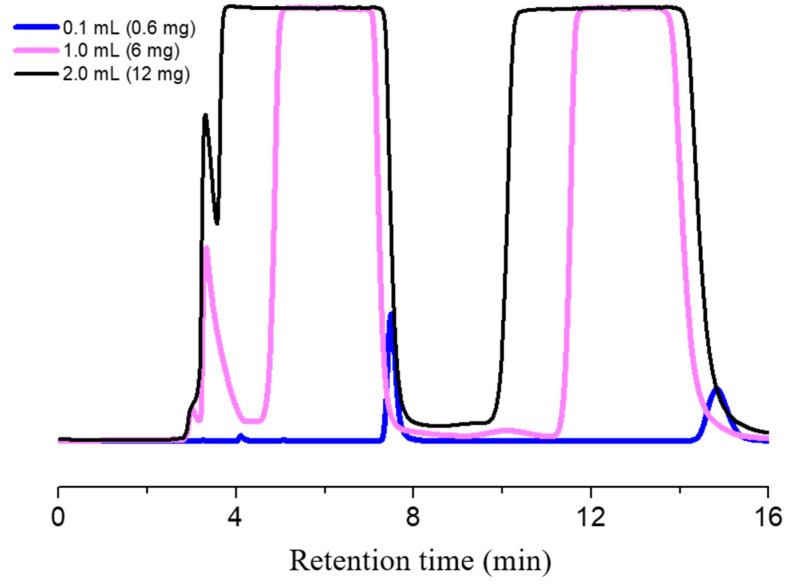
Loading study of **MBZ-OH** on the Chiralpak IG CSP. Chromatographic conditions: column, Chiralpak IG (250 mm × 10 mm, 5 μm); mobile phase, methanol; temperature, 25 °C; flow rate, 4.5 mL min^−1^; detection, UV at 254 nm.

As shown in Figure 6, peak splitting was prevented and two well-separated and symmetrical enantiomeric peaks were obtained by dissolving 60 mg of the racemic sample in 1 mL of DMF and diluting the mixture to 10 mL with methanol. The injection volume of the feed solution was gradually increased from 0.1 mL (corresponding to 0.6 mg) to 2.0 mL (corresponding to 12 mg). At the maximum loading limit, the peaks were separated again by the baseline.

Both fractions were collected with a quantitative yield of approximately 90% and showed an enantiomeric excess of more than 99%. Considering that the chromatographic run was completed in 15 min, approximately 21.5 mg of each enantiomer was collected per hour.

### 2.5. Chiroptical Properties and Absolute Configuration Assignment

A valuable method for determining the absolute configuration of organic molecules is to combine the study of the chiroptical properties of enantiopure or enantioenriched samples, such as electronic circular dichroism (ECD) or optical rotatory dispersion (ORD), with relevant theoretical calculations [31,32,33,34]. The analysis of the experimental data, based on the correspondences found by the chemical–quantum calculations, involves the development of several steps, starting with the execution of a thorough conformational search of the studied molecular structure (carried out with a progressive increase in the theoretical level of calculation), and continuing by subjecting the most populated and therefore most representative conformers to the simulation of the desired chiroptical properties, also taking into account, if appropriate, the solvent-induced effect. Finally, the last step is to track the ECD/ORD curves obtained by averaging the corresponding simulated spectra of the more representative conformers according to their respective previously estimated Boltzmann Distribution (**BD**).

**Figure 7:**
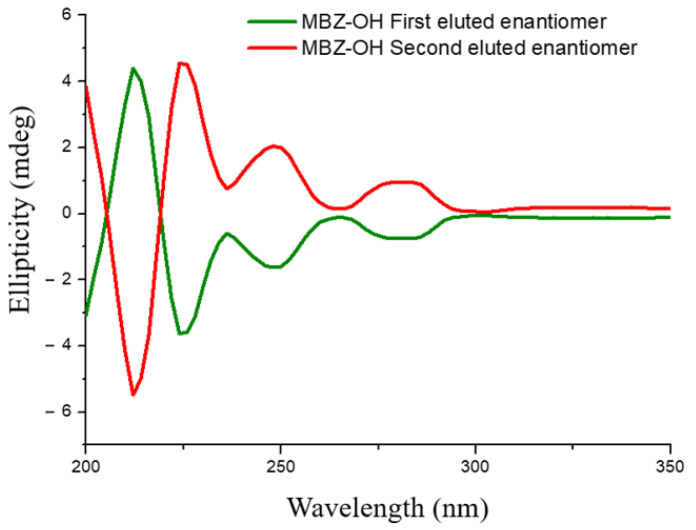
Experimental ECD spectra of **MBZ-OH** in methanol solution (green line refers to the first eluted enantiomer and the red line refers to the second eluted enantiomer).

**Figure 8:**
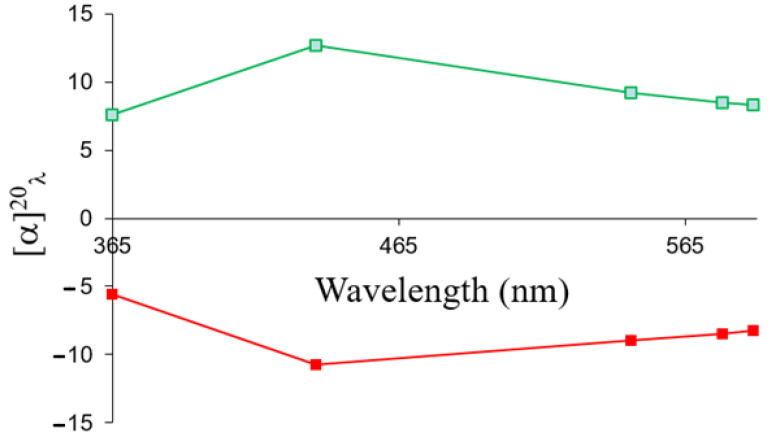
Experimental ORD curves of **MBZ-OH** in DMF solution (green line refers to the first eluted enantiomer and the red line refers to the second eluted enantiomer).

The experimental CD spectra and ORD curves of the antipodes of MBZ-OH (green and red solid lines in Figure 7 and Figure 8, respectively), isolated at a semi-preparative level, showed mirror profiles to confirm their enantiomeric relationships.

The CD spectrum of the first eluted enantiomer exhibited three consecutive negative bands and one positive band located at about 210 nm. The same enantiomer had a specific rotation value of +8.3 in DMF solution at 589 nm. The specific rotations of the second eluted enantiomer at the same wavelength was −8.2.

As shown in Figure 8, monotonic ORD curves were observed for two enantiomers at the five wavelengths studied (589, 578, 546, 436, and 365 nm). The maximum excursion of the specific rotation was recorded at 436 nm with +12.7 and −10.7 for the less- and more-retained enantiomers, respectively.

**Figure 9:**
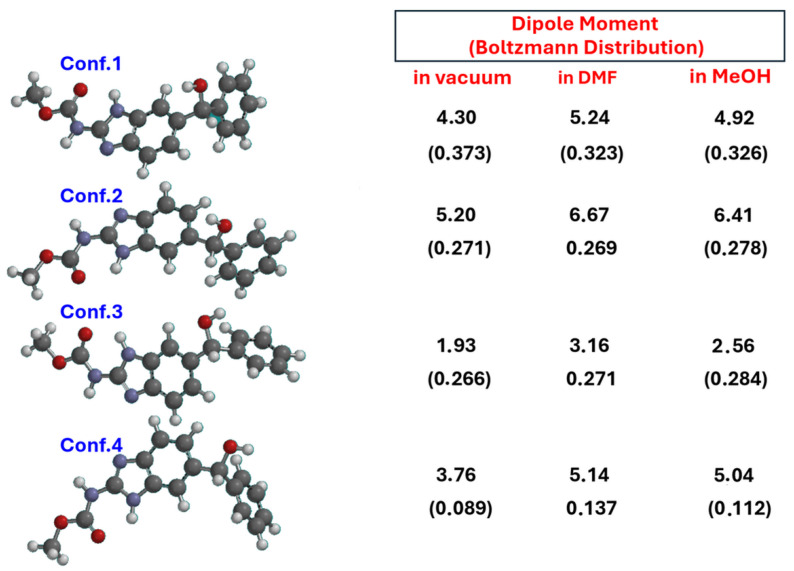
Computed dipole moment and Boltzmann Distribution of the four **Conf.1**–**4** more stable geometries of **MBZ-OH** obtained by computational search in vacuum, methanol and dimethylformamide.

According to the above-mentioned computational procedure, an extensive conformational search of the structure of (*R*)-**MBZ-OH** was carried out, followed by an appropriate selection of its energetically more representative conformations, which were then subjected to the evaluation of the ECD and ORD chiroptical properties (for more details see Section 3.5). In particular, four conformers of (*R*)-**MBZ-OH** were selected (**Conf.1**–**4**, shown in Figure 9) whose significant structural difference is due only to the free rotation around the two sigma bonds to which the chiral carbon is attached: (a) the hydroxyl group (~OH); (b) the benzimidazole-carbamate scaffold.

**Figure 10:**
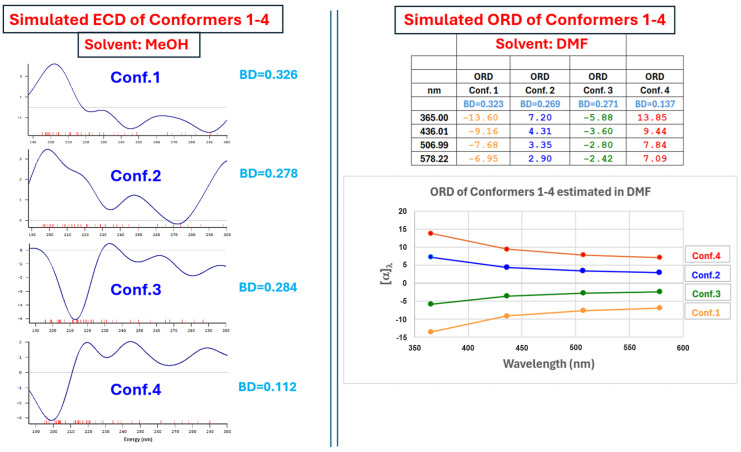
Simulation of the ECD and ORD curves of the four **Conf.1**–**4** more stable geometries of **MBZ-OH** obtained by computational search.

Simulation of the ECD and ORD curves of each conformer indicated that a change in orientation assumed by the ~OH group manifests a significant effect on the circular dichroism (the most intense ECD band centered between 200 and 210 nm assumes positive values when ~OH is oriented toward the benzimidazole-carbamate framework and negative values when ~OH is oriented toward the phenyl group), while the up–down flipping of the benzimidazole-carbamate portion leads to a determinant change in the optical rotation from negative (for **Conf.1** and **Conf.3**) to positive values (for **Conf.2** and **Conf.4**); all effects are visible in Figure 10.

**Figure 11:**
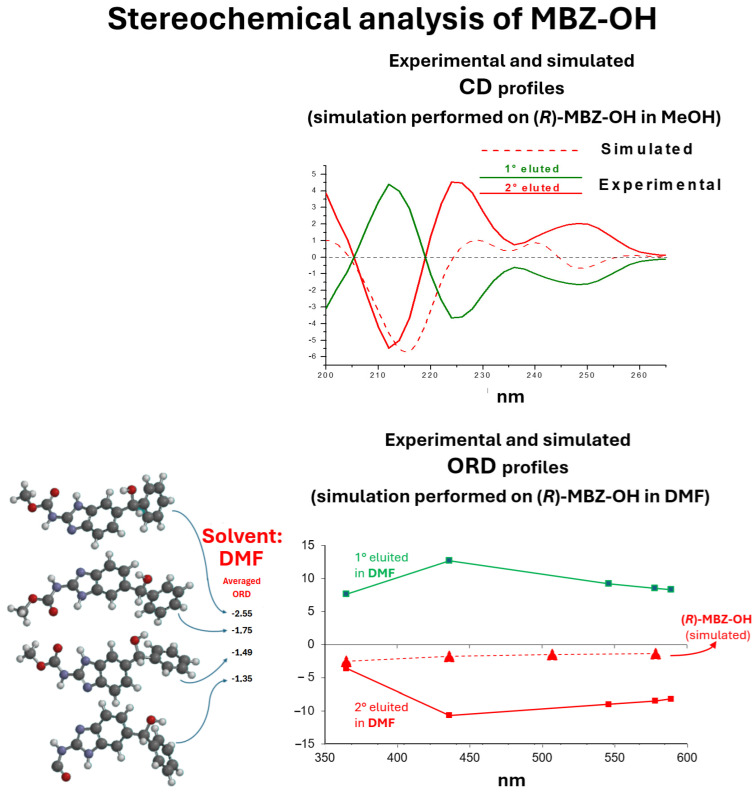
Stereochemical analysis of the first and second HPLC eluted enantiomers of **MBZ-OH**.

The quite different dipole moment (**dm**) and **BD**, assessed in vacuum, that characterize these four **Conf.1**–**4** structures, suggest that the relative stability of these latter may not undergo negligible modification as a function of the polarity of the solvent in which **MBZ-OH** is solubilized. This is indeed also computationally observed when their geometries are optimized, aside from in vacuum, in DMF (ε = 36.7) and in MeOH (ε = 36.7), and their **dm** and **BD** values were estimated in these solvents, as visible by the corresponding data reported in Figure 9. The assessed **dm** values provide evidence that all the **Conf.1**–**4** undergo little modifications, leaving a small increase in the polarity (of 0.9 and 1.2 debye for **Conf.1** and **Conf.3**, and of 1.5 and 1.4 debye for **Conf.2** and **Conf.4**), which is an effect that also has repercussions on the related **BD** values (in MeOH and DMF this leads the **Conf.3** becoming a little more populated than **Conf.2**). In this way, the simulated ECD and ORD curves, obtained by averaging the spectra estimated for **Conf.1**–**4** over the respective **BD** values, could also be affected in their trend. Therefore, taking into account all the above considerations, an evaluation of the configuration attributable to the **MBZ-OH** enantiomers eluted as first and second species from the enantioselective Chiralpak IG-3 column was carried out, with the limits of correctness suggested by the computational results discussed above (Figure 11).

In this way, the (*R*) configuration can be assigned to the second eluted **MBZ-OH** enantiomer and the (*S*) configuration to the first eluted one.

## 3. Materials and Methods

### 3.1. Chemistry

All the solvents and reagents were used as supplied by Sigma-Aldrich (Milan, Italy). The mixtures of solvents are reported as *volume/volume* ratios. The melting points were determined on a Stuart^®^ melting point apparatus SMP1 and are uncorrected (temperatures are reported in °C). The chemical and structural characterization was accomplished through ^1^H/^13^C-NMR analysis. ^1^H and ^13^C-NMR spectra were recorded at 400 MHz and 101 MHz, respectively, on a Bruker spectrometer, using DMSO-*d*_6_ as the solvent at room temperature. **MBZ-OH** was studied using NMR at the final concentration of ~20 mg/mL. ^1^H and ^13^C chemical shifts are reported as *δ* units (parts per million) relative to the solvent signal. ^1^H spectra are described as follows: *δ*_H_ (spectrometer frequency, solvent): chemical shift/ppm (multiplicity, J-coupling constant(s) in Hertz (Hz), number of protons, assignment). ^13^C spectra are described as follows: *δ*_C_ (spectrometer frequency, solvent): chemical shift/ppm (assignment) and are fully proton-decoupled. Multiplets are abbreviated as follows: br—broad; s—singlet; d—doublet; t—triplet; m—multiplet. The exchangeable protons (OH, NH) were evaluated by the addition of deuterium oxide (D_2_O). MestreNova was employed for processing and analyses of the NMR data. Preparative chromatography was carried out employing silica gel (high purity grade, pore size 60 Å, 230–400 mesh particle size). The reaction and purification were checked by thin layer chromatography (TLC) performed on 0.2 mm thick silica gel–aluminium-backed plates (60 F254). Spot visualization was performed under short- and long-wavelength (254 and 365 nm, respectively) ultra-violet irradiation. Where given, systematic compound names were generated by ChemBioDraw Ultra 14.0 following IUPAC conventions. High-resolution mass spectrometry (HRMS) analysis was performed using a matrix-assisted laser desorption/ionization time of flight (MALDI-TOF/TOF) instrument (Bruker UltrafleXtreme, Bremen, Germany), used in the positive, reflector mode. A saturated solution of α-2-cyano-4-hydroxycinnamic acid (CHCA) dissolved in acetonitrile, water, and trifluoroacetic acid mixed in a 50:50:1 ratio was used as the substrate. Second eluted **MBZ-OH** was dissolved in methanol at a concentration of 0.01 µg mL^−1^; 1 μL of this solution was pipetted on the MALDI target board, followed by 1 μL of the substrate solution (matrix). The conditions of the time-of-flight mass spectrometer were set as follows: Reflection mode, excitation voltage 20 KV, Scanning range 100–1000 Da. The signals of the matrix were used as internal standards for analysis calibration.

### 3.2. Synthesis of ***MBZ-OH***

To a stirring suspension of mebendazole (MBZ, 0.001 eq., 0.3 g) in dry methanol (40 mL) at room temperature was added an excess of sodium borohydride (NaBH_4_, 0.010 eq., 0.38 g) portionwise. The reaction was monitored by TLC and upon completion (8 h) was quenched with 2 N HCl. The aqueous phase was extracted with ethyl acetate (5 × 30 mL), and the organic layers were reunited, dried over sodium sulfate, and concentrated in vacuo. The crude product was purified by column chromatography on silica gel employing as mobile phase a mixture of dichloromethane/methanol in a 20:1 (*v/v*) ratio. The title compound was a white solid (84% yield); mp > 300 °C. ^1^H-NMR (400 MHz, DMSO-*d*_6_) *δ* 3.73 (s, 3H, CH_3_), 5.75 (d, *J* = 4.0 Hz, 1H, CH-OH, D_2_O exch.), 5.80 (d, *J* = 4.0 Hz, 1H, CH-OH), 7.06–7.09 (m, 1H, CH_Ar_), 7.18 (t, *J* = 7.2 Hz, 1H, CH_Ar_), 7.28 (t, *J* = 7.7 Hz, 3H, CH_Ar_), 7.36–7.41 (m, 3H, CH_Ar_), 11.64 (s, 2H, NH_benzimidazole_ + NH_carbamate_, D_2_O exch.). ^13^C-NMR (101 MHz, DMSO-*d*_6_) *δ* 52.8 (CH_3_), 75.1 (CH-OH), 111.7 (Ar), 113.4 (Ar), 120.4 (Ar), 126.7 (2 × Ar), 126.9 (Ar), 128.4 (2 × Ar), 135.3 (Ar), 136.0 (Ar), 139.4 (Ar), 146.8 (Ar), 148.4 (Ar), 155.8 (NHC=O). HRMS (+) *m/z* 298.1186 [MH]^+^ (calculated for C_16_H_15_N_3_O_3_, 298.1179; Δm = 2.34 ppm).

### 3.3. Instruments and Chromatographic Conditions

Analytical-scale separation of enantiomers of **MBZ-OH** was performed on a UHPLC Jasco LC-4000 (Jasco, Tokyo, Japan). This instrument included a binary pumping system with a maximum flow rate of 2 mL min^−1^, an autosampler with an injection loop volume of 20 μL (used in partial loop mode), an MD-4010 photodiode array detector with a 16 μL internal flow cell, and a column oven. Data acquisition, data processing, and instrument control were performed using Jasco ChomNAV 2.04.00 software.

For semi-preparative resolutions, the HPLC apparatus consisted of a Perkin-Elmer pump (LC 2000 series) (Norwalk, CT, USA), a Rheodyne injector (Cotati, CA, USA), a 3000 μL sample loop, a detector (UV Waters 484, Waters Corporation, Milford, MA, USA) and a Perkin-Elmer LC 101 HPLC thermostat (Sunnyvale, CA, USA). Data acquisition and data processing were performed using ClarityTM 7.1.0.151 software from DataApex (Prague, Czech Republic).

The columns used in this study were Chiralpak IG-3 (100 mm × 4.6 mm, 3.0 μm) and Chiralpak IG (250 mm × 10 mm, 5 μm) from Chiral Technologies Europe (Illkirch-Graffenstaden, France). HPLC-grade solvents of methanol, ethanol and water were purchased from Sigma-Aldrich (Milan, Italy).

Fresh standard solutions of the synthesized **MBZ-OH** were prepared by dissolving the analytes in methanol at a concentration of approximately 1.0 mg mL^−1^. The injection volume was 10 μL. Solvents and samples were filtered through 0.22 μm filters. The hold-up time was estimated using 1,3,5-tri-tert-butylbenzene as a marker.

ECD spectra of enantiomers of **MBZ-OH** in methanol solution were measured in a 0.1 cm path length quartz cell at 25 °C using a Jasco model J-700 spectropolarimeter. The spectra were averaged over four instrumental scans and the intensities are expressed as ellipticity values (mdeg).

Specific rotations of the enantiomers of **MBZ-OH** in dimethylformamide solution (concentration 0.5 g/100 mL) were measured at 589, 578, 546, 436 and 365 nm by a PerkinElmer polarimeter model 241 equipped with Na/Hg lamps. The volume of the cell was 1 mL, and the optical path was 10 cm. The system was set at a temperature of 20 °C.

### 3.4. Semi-Preparative Separation of ***MBZ-OH*** Enantiomers

Semi-preparative enantioseparations were performed using a Chiralpak IG column (250 mm × 10 mm, 5 μm) and pure methanol as the mobile phase. The temperature and flow rate were set at 25 °C and 4.5 mL min^−1^, respectively. After injecting 2.0 mL of the sample solution (obtained by dissolving 60 mg of racemic sample in 1 mL of DMF and diluting to 10 mL with methanol) (equivalent to 12 mg), two fractions were collected. Evaporation of the pooled fractions yielded a white solid. The enantiomeric excess of the collected enantiomers was evaluated by HPLC using the Chiralpak IG-3 analytical column (100 mm × 4.6 mm, 3 μm) and the same mobile phase used in the mg scale resolution. The first eluted enantiomer of MBZ-OH had a specific rotation value of +8.3 in DMF solution at 589 nm, while the second eluted enantiomer had a specific rotation value of −8.2 at the same wavelength. The enantiomeric excess of both enantiomers was higher than 99%. The quantitative yield of HPLC semi-preparative resolution was approximately 90% for both enantiomers.

### 3.5. Conformational Analysis and Prediction of Chiroptical Properties

Before using the predicted chiroptical properties to interpret the corresponding experimental data, the predominant conformations of (*R*)-**MBZ-OH** were searched by molecular mechanics calculations (force field: MMFF), according to the systematic algorithm implemented in the computer program SPARTAN 10 v.1.1.0 (Wavefunction Inc., 18401 Von Karman Avenue, Suite 370, Irvine, CA 92612, USA). The employed conditions adopted were as follows: (i) all the rotatable bonds varied; (ii) 40 kJ·mol^−1^ was the maximum energy gap from the lowest energy geometry imposed for the kept conformations; (iii) R^2^ ≥ 0.9 was the criterion used to define conformers as duplicates in the analysis of similarity between conformations. Starting from a geometry of the (*R*) configuration of compound **MBZ-OH**, the molecular mechanic conformational search afforded eight **Conf.1**–**Conf.8** different geometries which, afterward, were further optimized, before at the Hartree Fook 3-21G level of theory, next at the higher Density Functional B3LYP/6-31G* level of theory, in this case discarding four conformations that resulted characterized by an energy difference from the global minimum of more than 3 kcal. The final relative differences in the energy of conformers **Conf.2**, **Conf.3**, and **Conf.4** with respect to the global minimum (GM) **Conf.1** (0.00 kcal mol^−1^) were as follows: **Conf.2** = 0.23, **Conf.3** = 0.23, **Conf.4** = 0.89 kcal mol^−1^. At 25 °C, these energy gaps correspond to the following percentages of Boltzmann Distribution (**BD**): 38.9% (**Conf.1**), 26.3% (**Conf.2**), 26.2% (**Conf.3**) and 8.6% (**Conf.4**). According to these data, the simulation of ECD spectra and ORD curves, performed through the algorithms implemented in the Amsterdam Density Functional (ADF) package v. 2007.01, were performed on all these geometries. The options set to carry out the ECD calculations were as follows: the single point at the BLYP level of theory, employing the TZP Large Core basis set; vacuum, MeOH as the solvent; 45 singlet-only excitations; diagonalization method: Davidson; velocity representation; scaling factor 1.00; peak width 20.0. The final simulated ECD spectrum, obtained by averaging the pertinent simulated spectra for **Conf.1**–**4** according to their Boltzmann populations, is reported in Figure 11, superimposed on the experimental one relevant to the isolated enantiomers of **MBZ-OH**. The options set to carry out the ORD calculations were as follows: the single point at the BLYP level of theory, employing the DZP Large Core basis set; DMF as the solvent; optical rotation with 4 frequencies, from 3650 to 5780 Angstrom. The final simulated ORD curve, obtained by averaging the pertinent simulated ORD for **Conf.1**–**4** according to their Boltzmann populations, is reported in Figure 11, superimposed on the experimental ones of both the isolated enantiomers of **MBZ-OH**.

## 4. Conclusions

In conclusion, this work demonstrated that immobilized-type ACMPC-based CSPs are effective chiral chromatographic materials for the resolution of **MBZ-OH**. The columns packed with this type of CSP provided remarkable enantioseparation under polar organic mode as well as HILIC and RP conditions. This versatility can potentially be exploited to set up HPLC protocols capable of (i) assessing the stereochemical course of the reduction of **MBZ** to **MBZ-OH** in biological fluids and (ii) collecting enantiopure forms at the multi-milligram scale.

In our opinion, the results of the present study will trigger the evaluation of individual enantiomers of MBZ-OH as lead compounds in the drug discovery process.

## Figures and Tables

**Table 1:** Retention factor (*k*_1_) for the first eluting enantiomer, enantioseparation (*α*) and resolution (*Rs*) factors of **MBZ-OH** in polar organic conditions. Chromatographic conditions: column, Chiralpak IG-3 (100 mm × 4.6 mm, 3 μm); flow rate, 1 mL min^−1^; detection, UV at 220 nm.

Mobile Phase	Temperature (°C)	*k*_1_	*α*	*Rs*
Ethanol	20	1.96	2.55	4.84
	25	1.86	2.44	5.26
	30	1.75	2.33	5.60
	35	1.69	2.19	5.73
	40	1.55	2.13	5.88
Methanol	20	2.07	2.51	5.79
	25	2.01	2.38	6.13
	30	1.92	2.28	5.07
	35	1.85	2.18	5.08
	40	1.77	2.10	5.01
Acetonitrile	25	Not eluted after 30 min		

## Data Availability

Data will be made available on request.
